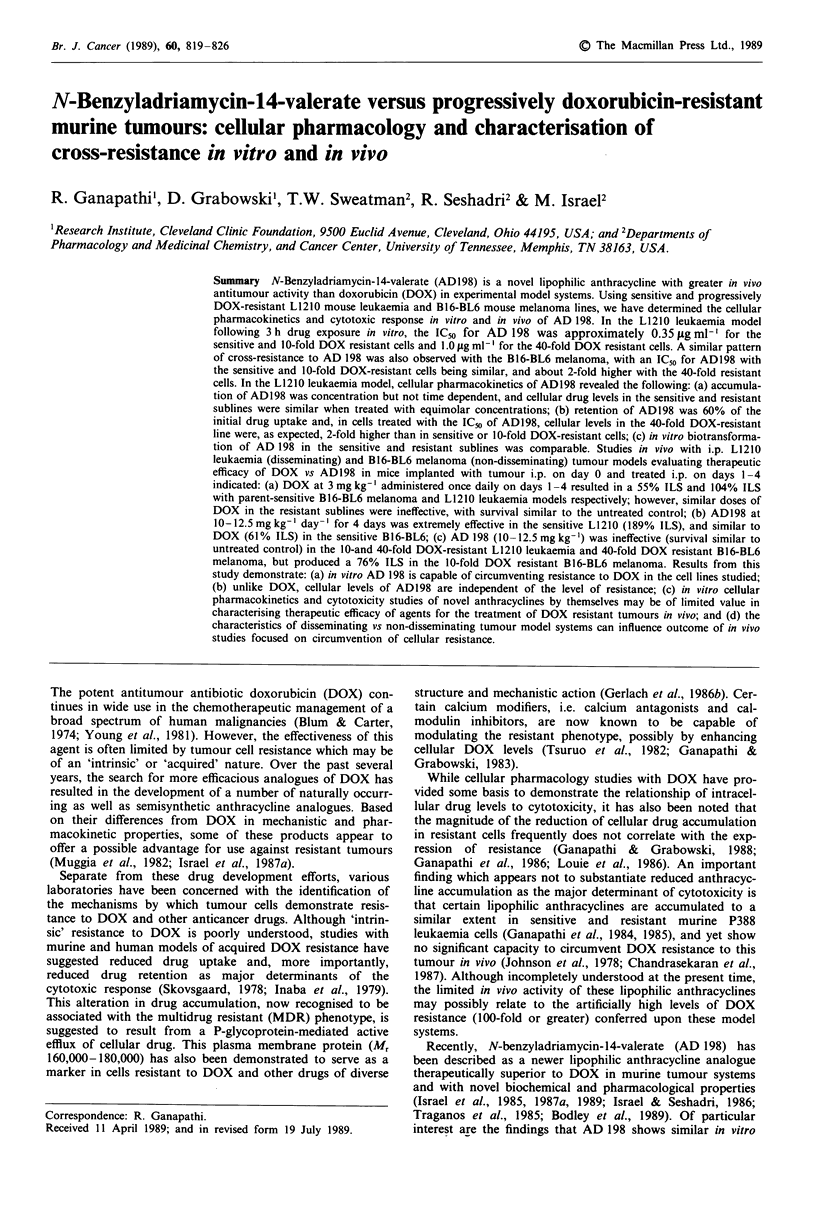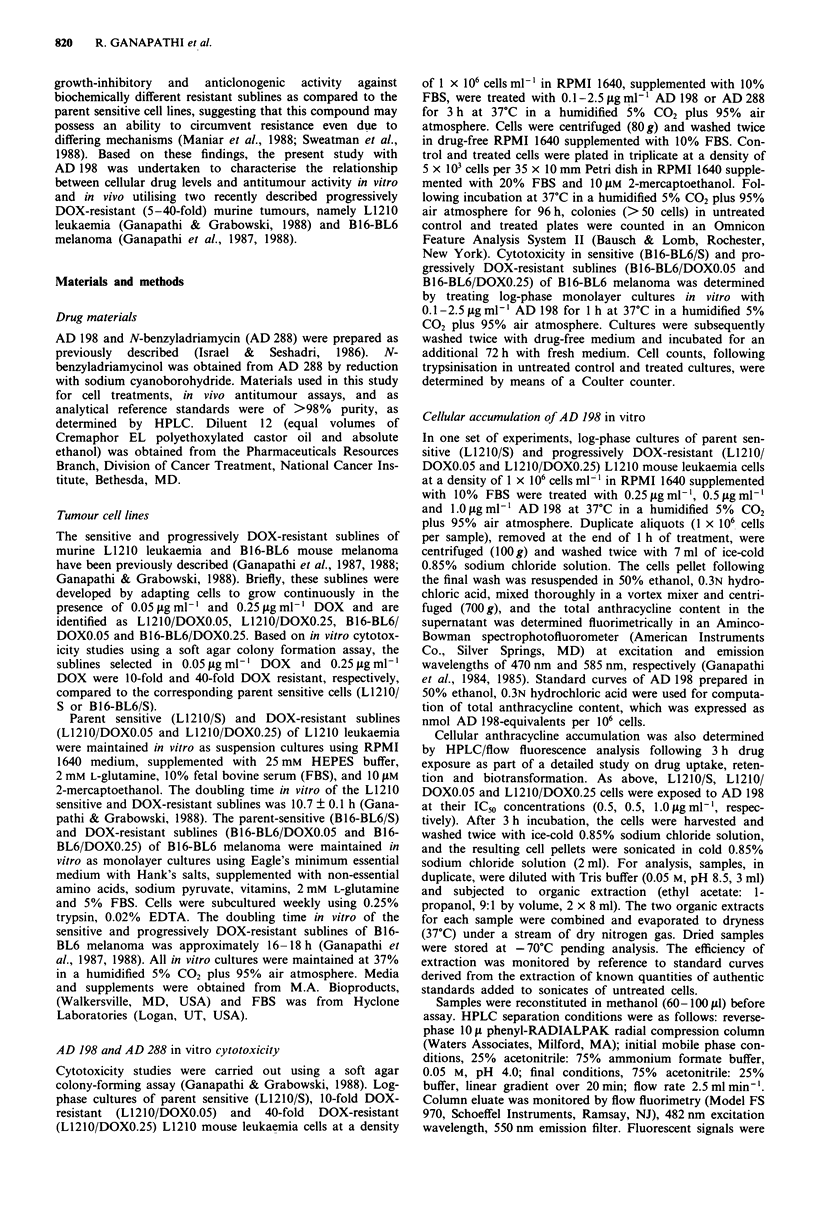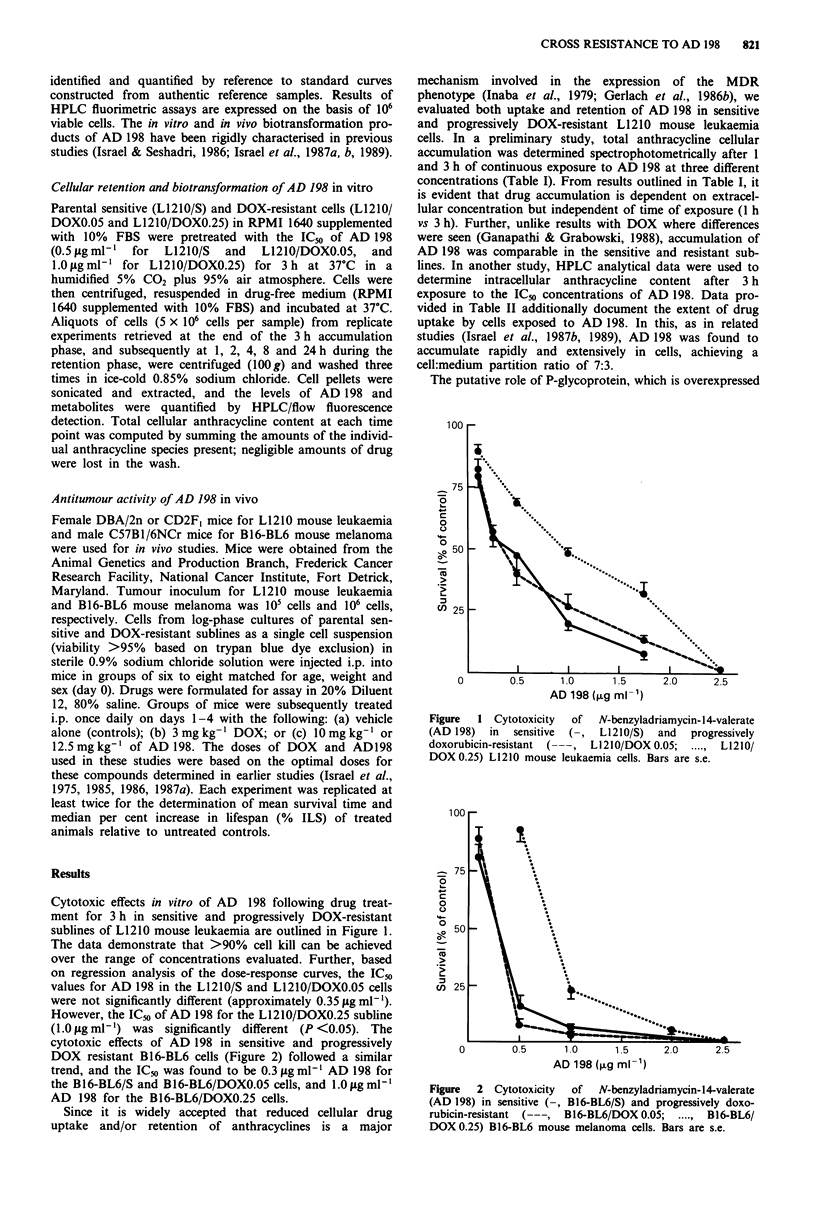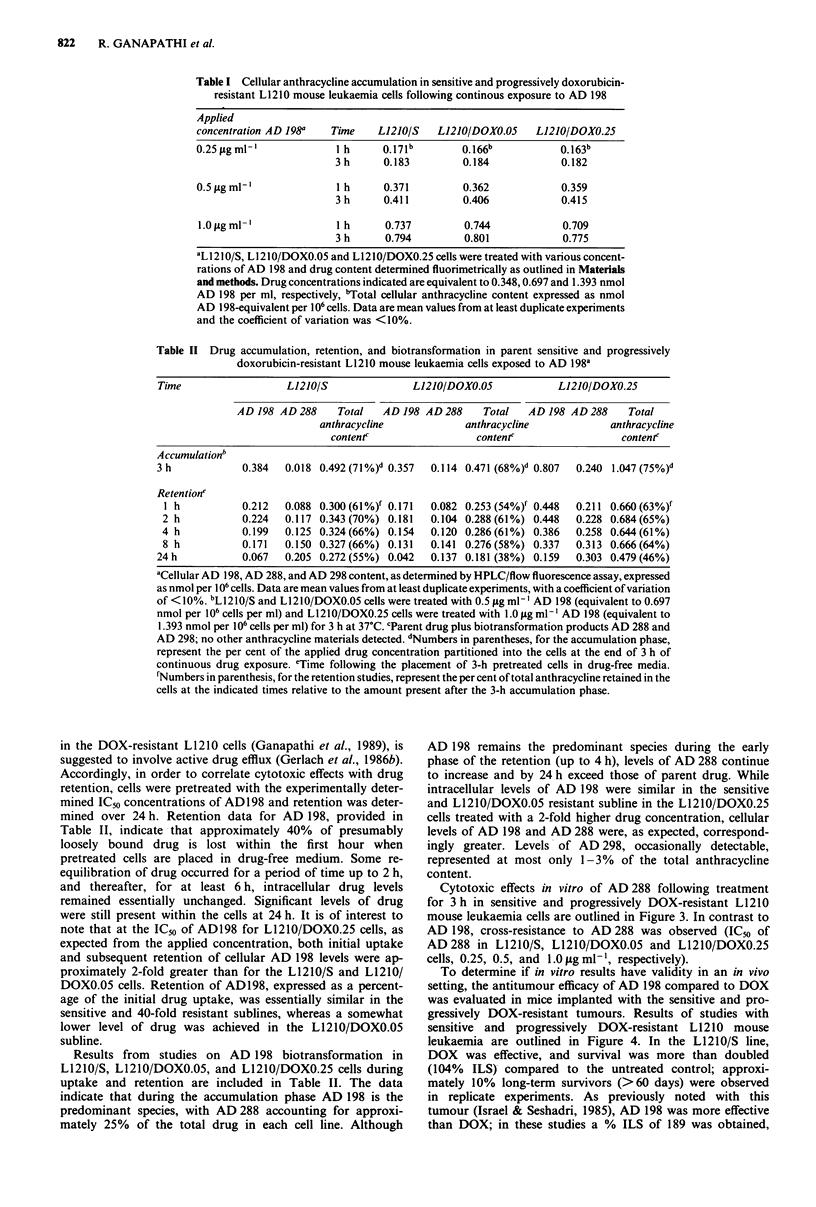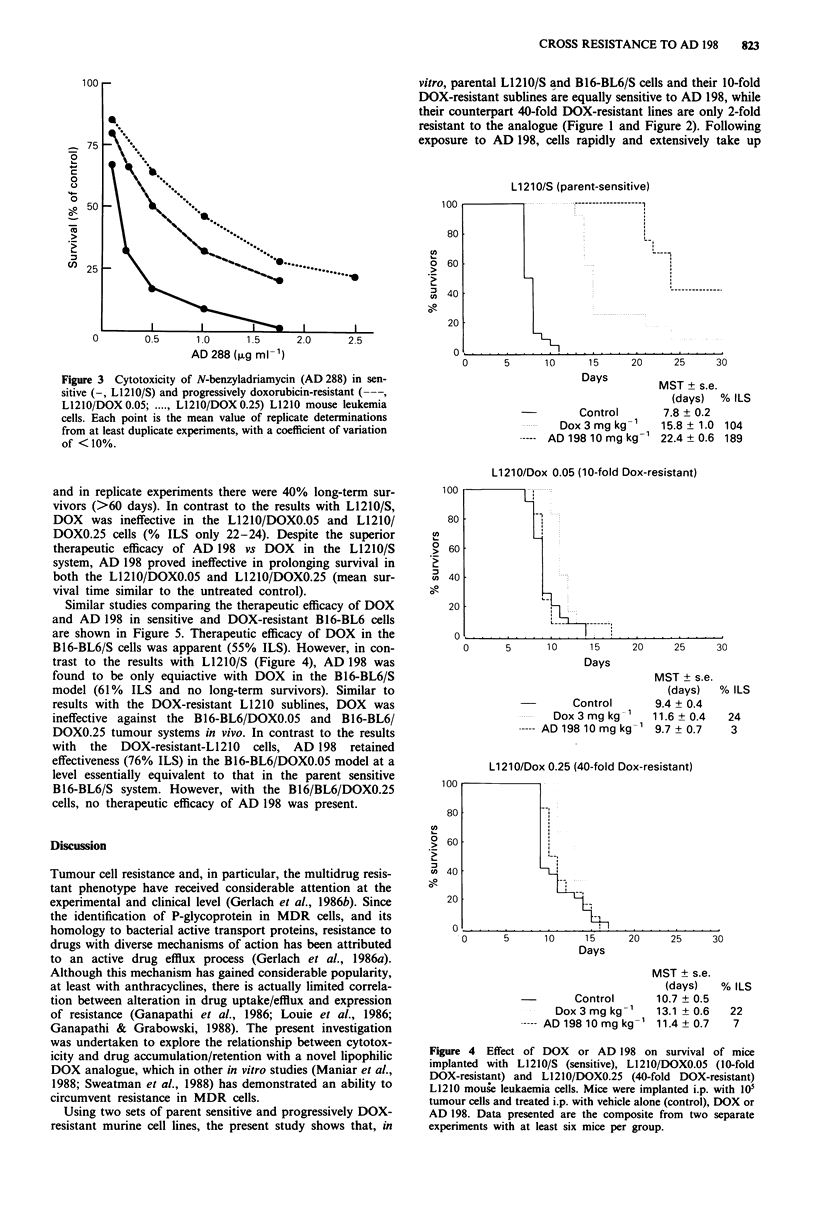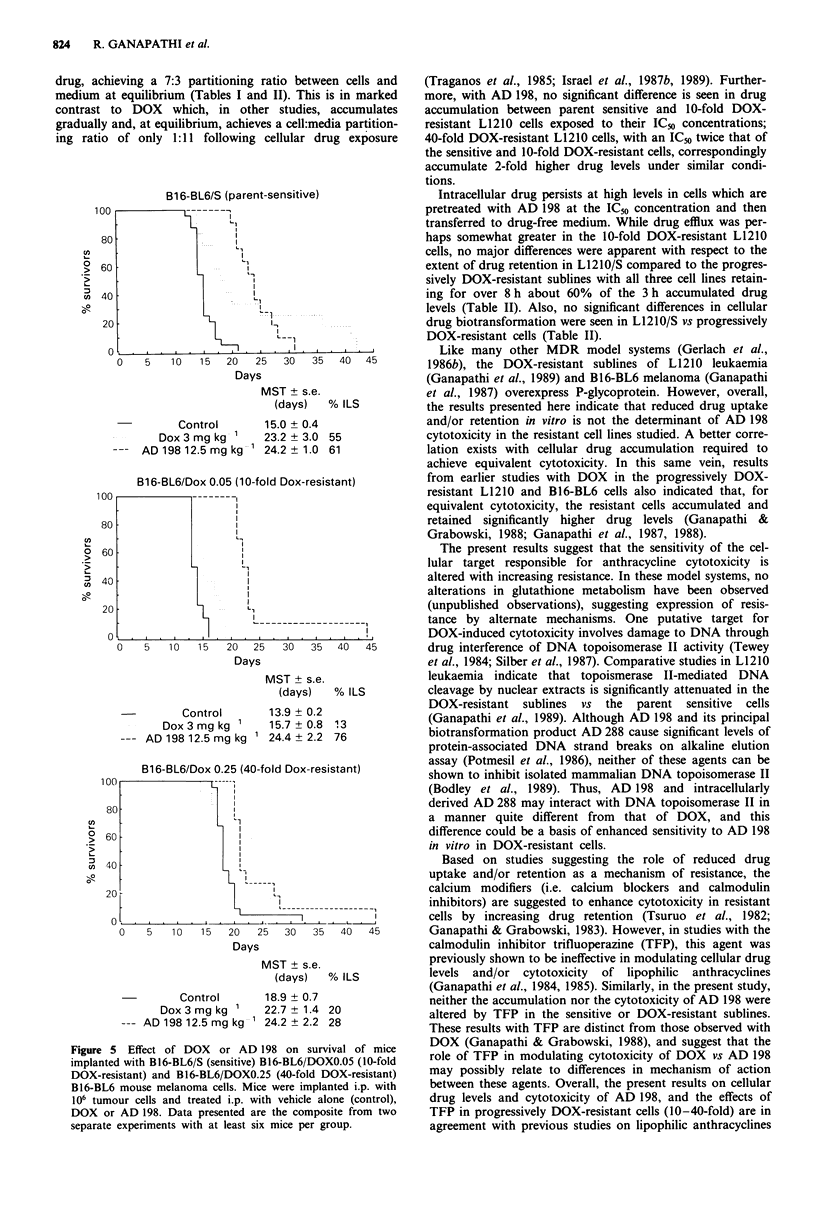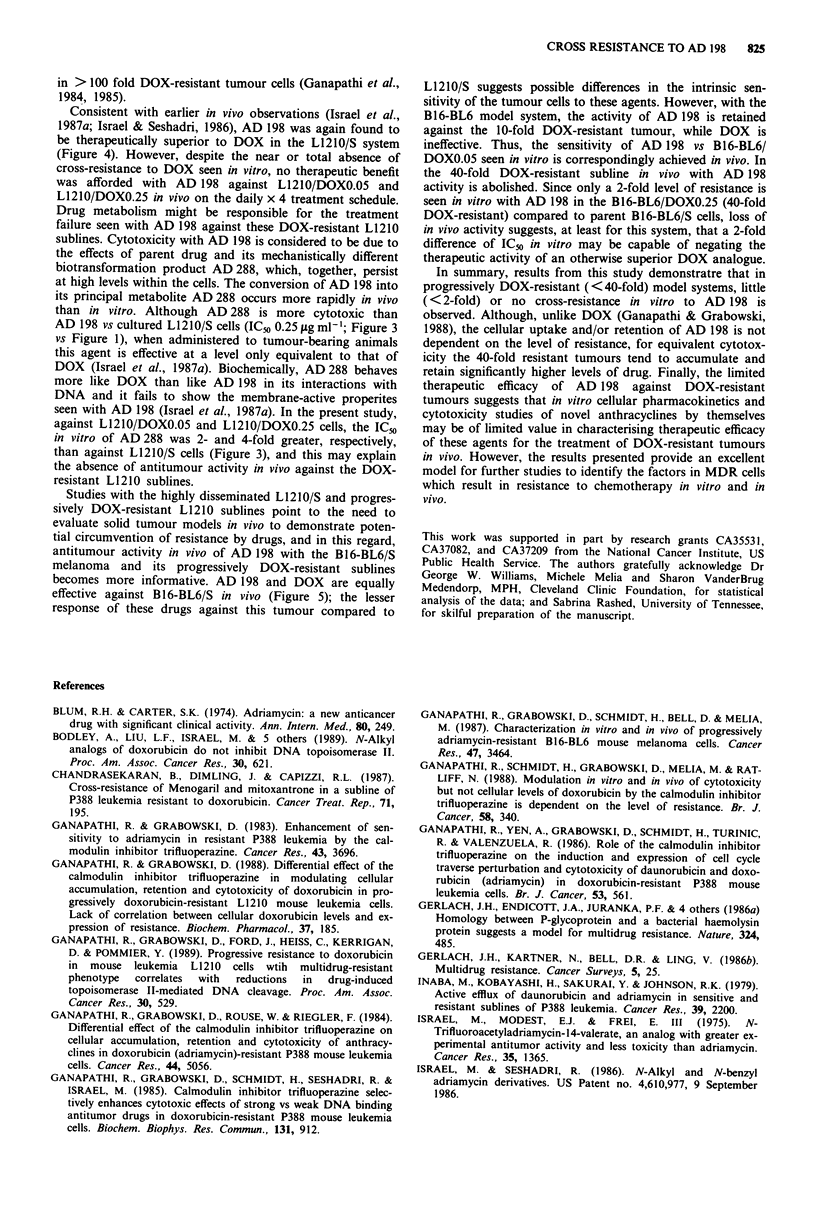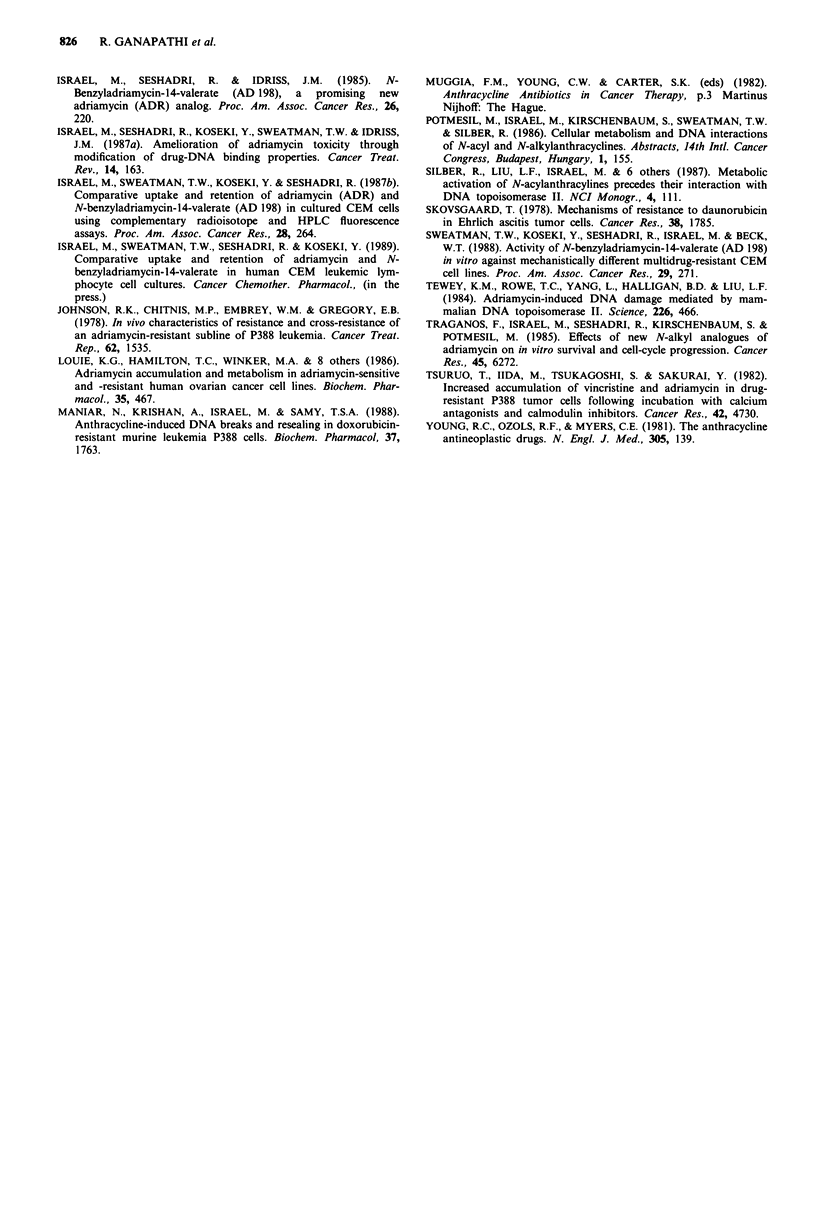# N-benzyladriamycin-14-valerate versus progressively doxorubicin-resistant murine tumours: cellular pharmacology and characterisation of cross-resistance in vitro and in vivo.

**DOI:** 10.1038/bjc.1989.373

**Published:** 1989-12

**Authors:** R. Ganapathi, D. Grabowski, T. W. Sweatman, R. Seshadri, M. Israel

**Affiliations:** Research Institute, Cleveland Clinic Foundation, Ohio 44195.

## Abstract

N-Benzyladriamycin-14-valerate (AD198) is a novel lipophilic anthracycline with greater in vivo antitumour activity than doxorubicin (DOX) in experimental model systems. Using sensitive and progressively DOX-resistant L1210 mouse leukaemia and B16-BL6 mouse melanoma lines, we have determined the cellular pharmacokinetics and cytotoxic response in vitro and in vivo of AD198. In the L1210 leukaemia model following 3 h drug exposure in vitro, the IC50 for AD198 was approximately 0.35 microgram ml-1 for the sensitive and 10-fold DOX resistant cells and 1.0 microgram ml-1 for the 40-fold DOX resistant cells. A similar pattern of cross-resistance to AD198 was also observed with the B16-BL6 melanoma, with and IC50 for AD198 with the sensitive and 10-fold DOX-resistant cells being similar, and about 2-fold higher with the 40-fold resistant cells. In the L1210 leukaemia model, cellular pharmacokinetics of AD198 revealed the following: (a) accumulation of AD198 was concentration but not time dependent, and cellular drug levels in the sensitive and resistant sublines were similar when treated with equimolar concentrations; (b) retention of AD 198 was 60% of the initial drug uptake and, in cells treated with the IC50 of AD198, cellular levels in the 40-fold DOX-resistant line were, as expected, 2-fold higher than in sensitive or 10-fold DOX-resistant cells; (c) in vitro biotransformation of AD 198 in the sensitive and resistant sublines was comparable. Studies in vivo with i.p. L1210 leukaemia (disseminating) and B16-BL6 melanoma (non-disseminating) tumour models evaluating therapeutic efficacy of DOX vs AD 198 in mice implanted with tumour i.p. on day 0 and treated i.p. on days 1-4 indicated: (a) DOX at 3 mg kg-1 administered once daily on days 1-4 resulted in a 55% ILS and 104% ILS with parent-sensitive B16-BL6 melanoma and L1210 leukaemia models respectively; however, similar doses of DOX in the resistant sublines were ineffective, with survival similar to the untreated control; (b) AD198 at 10-12.5 mg kg-1 day-1 for 4 days was extremely effective in the sensitive L1210 (189% ILS), and similar to DOX (61% ILS) in the sensitive B16-BL6; (c) AD198 (10-12.5 mg kg-1) was ineffective (survival similar to untreated control) in the 10-and 40-fold DOX-resistant L1210 leukaemia and 40-fold DOX resistant B16-BL6 melanoma, but produced a 76% ILS in the 10-fold DOX resistant B16-BL6 melanoma.(ABSTRACT TRUNCATED AT 400 WORDS)


Br. J. Cancer (1989), 60, 819-826 © The Macmillan Press Ltd., 1989

### *N*-Benzyladriamycin-14-valerate versus progressively doxorubicin-resistant murine tumours: cellular pharmacology and characterisation of cross-resistance *in vitro* and *in vivo*

R. Ganapathi[1], D. Grabowski[1], T.W. Sweatman[2], R. Seshadri[2] & M. Israel[2]

[1]*Research Institute, Cleveland Clinic Foundation, 9500 Euclid Avenue, Cleveland, Ohio 44195, USA; and* [2]*Departments of Pharmacology and Medicinal Chemistry, and Cancer Center, University of Tennessee, Memphis, TN 38163, USA.*

**Summary** *N*-Benzyladriamycin-14-valerate (AD198) is a novel lipophilic anthracycline with greater *in vivo* antitumour activity than doxorubicin (DOX) in experimental model systems. Using sensitive and progressively DOX-resistant L1210 mouse leukaemia and B16-BL6 mouse melanoma lines, we have determined the cellular pharmacokinetics and cytotoxic response *in vitro* and *in vivo* of AD 198. In the L1210 leukaemia model following 3 h drug exposure *in vitro*, the $IC_{50}$ for AD 198 was approximately 0.35 $\mu g\,ml^{-1}$ for the sensitive and 10-fold DOX resistant cells and 1.0 $\mu g\,ml^{-1}$ for the 40-fold DOX resistant cells. A similar pattern of cross-resistance to AD 198 was also observed with the B16-BL6 melanoma, with an $IC_{50}$ for AD198 with the sensitive and 10-fold DOX-resistant cells being similar, and about 2-fold higher with the 40-fold resistant cells. In the L1210 leukaemia model, cellular pharmacokinetics of AD198 revealed the following: (a) accumulation of AD198 was concentration but not time dependent, and cellular drug levels in the sensitive and resistant sublines were similar when treated with equimolar concentrations; (b) retention of AD198 was 60% of the initial drug uptake and, in cells treated with the $IC_{50}$ of AD198, cellular levels in the 40-fold DOX-resistant line were, as expected, 2-fold higher than in sensitive or 10-fold DOX-resistant cells; (c) *in vitro* biotransformation of AD 198 in the sensitive and resistant sublines was comparable. Studies *in vivo* with i.p. L1210 leukaemia (disseminating) and B16-BL6 melanoma (non-disseminating) tumour models evaluating therapeutic efficacy of DOX *vs* AD198 in mice implanted with tumour i.p. on day 0 and treated i.p. on days 1-4 indicated: (a) DOX at 3 $mg\,kg^{-1}$ administered once daily on days 1-4 resulted in a 55% ILS and 104% ILS with parent-sensitive B16-BL6 melanoma and L1210 leukaemia models respectively; however, similar doses of DOX in the resistant sublines were ineffective, with survival similar to the untreated control; (b) AD198 at 10-12.5 $mg\,kg^{-1}\,day^{-1}$ for 4 days was extremely effective in the sensitive L1210 (189% ILS), and similar to DOX (61% ILS) in the sensitive B16-BL6; (c) AD 198 (10-12.5 $mg\,kg^{-1}$) was ineffective (survival similar to untreated control) in the 10-and 40-fold DOX-resistant L1210 leukaemia and 40-fold DOX resistant B16-BL6 melanoma, but produced a 76% ILS in the 10-fold DOX resistant B16-BL6 melanoma. Results from this study demonstrate: (a) *in vitro* AD 198 is capable of circumventing resistance to DOX in the cell lines studied; (b) unlike DOX, cellular levels of AD198 are independent of the level of resistance; (c) *in vitro* cellular pharmacokinetics and cytotoxicity studies of novel anthracyclines by themselves may be of limited value in characterising therapeutic efficacy of agents for the treatment of DOX resistant tumours *in vivo*; and (d) the characteristics of disseminating *vs* non-disseminating tumour model systems can influence outcome of *in vivo* studies focused on circumvention of cellular resistance.

The potent antitumour antibiotic doxorubicin (DOX) continues in wide use in the chemotherapeutic management of a broad spectrum of human malignancies (Blum & Carter, 1974; Young *et al.*, 1981). However, the effectiveness of this agent is often limited by tumour cell resistance which may be of an 'intrinsic' or 'acquired' nature. Over the past several years, the search for more efficacious analogues of DOX has resulted in the development of a number of naturally occurring as well as semisynthetic anthracycline analogues. Based on their differences from DOX in mechanistic and pharmacokinetic properties, some of these products appear to offer a possible advantage for use against resistant tumours (Muggia *et al.*, 1982; Israel *et al.*, 1987*a*).

Separate from these drug development efforts, various laboratories have been concerned with the identification of the mechanisms by which tumour cells demonstrate resistance to DOX and other anticancer drugs. Although 'intrinsic' resistance to DOX is poorly understood, studies with murine and human models of acquired DOX resistance have suggested reduced drug uptake and, more importantly, reduced drug retention as major determinants of the cytotoxic response (Skovsgaard, 1978; Inaba *et al.*, 1979). This alteration in drug accumulation, now recognised to be associated with the multidrug resistant (MDR) phenotype, is suggested to result from a P-glycoprotein-mediated active efflux of cellular drug. This plasma membrane protein ($M_r$ 160,000-180,000) has also been demonstrated to serve as a marker in cells resistant to DOX and other drugs of diverse structure and mechanistic action (Gerlach *et al.*, 1986*b*). Certain calcium modifiers, i.e. calcium antagonists and calmodulin inhibitors, are now known to be capable of modulating the resistant phenotype, possibly by enhancing cellular DOX levels (Tsuruo *et al.*, 1982; Ganapathi & Grabowski, 1983).

While cellular pharmacology studies with DOX have provided some basis to demonstrate the relationship of intracellular drug levels to cytotoxicity, it has also been noted that the magnitude of the reduction of cellular drug accumulation in resistant cells frequently does not correlate with the expression of resistance (Ganapathi & Grabowski, 1988; Ganapathi *et al.*, 1986; Louie *et al.*, 1986). An important finding which appears not to substantiate reduced anthracycline accumulation as the major determinant of cytotoxicity is that certain lipophilic anthracyclines are accumulated to a similar extent in sensitive and resistant murine P388 leukaemia cells (Ganapathi *et al.*, 1984, 1985), and yet show no significant capacity to circumvent DOX resistance to this tumour *in vivo* (Johnson *et al.*, 1978; Chandrasekaran *et al.*, 1987). Although incompletely understood at the present time, the limited *in vivo* activity of these lipophilic anthracyclines may possibly relate to the artificially high levels of DOX resistance (100-fold or greater) conferred upon these model systems.

Recently, *N*-benzyladriamycin-14-valerate (AD 198) has been described as a newer lipophilic anthracycline analogue therapeutically superior to DOX in murine tumour systems and with novel biochemical and pharmacological properties (Israel *et al.*, 1985, 1987*a*, 1989; Israel & Seshadri, 1986; Traganos *et al.*, 1985; Bodley *et al.*, 1989). Of particular interest are the findings that AD 198 shows similar *in vitro*

Correspondence: R. Ganapathi.
Received 11 April 1989; and in revised form 19 July 1989.

growth-inhibitory and anticlonogenic activity against biochemically different resistant sublines as compared to the parent sensitive cell lines, suggesting that this compound may possess an ability to circumvent resistance even due to differing mechanisms (Maniar et al., 1988; Sweatman et al., 1988). Based on these findings, the present study with AD 198 was undertaken to characterise the relationship between cellular drug levels and antitumour activity *in vitro* and *in vivo* utilising two recently described progressively DOX-resistant (5–40-fold) murine tumours, namely L1210 leukaemia (Ganapathi & Grabowski, 1988) and B16-BL6 melanoma (Ganapathi et al., 1987, 1988).

### Materials and methods

### *Drug materials*

AD 198 and *N*-benzyladriamycin (AD 288) were prepared as previously described (Israel & Seshadri, 1986). *N*-benzyladriamycinol was obtained from AD 288 by reduction with sodium cyanoborohydride. Materials used in this study for cell treatments, *in vivo* antitumour assays, and as analytical reference standards were of >98% purity, as determined by HPLC. Diluent 12 (equal volumes of Cremaphor EL polyethoxylated castor oil and absolute ethanol) was obtained from the Pharmaceuticals Resources Branch, Division of Cancer Treatment, National Cancer Institute, Bethesda, MD.

### *Tumour cell lines*

The sensitive and progressively DOX-resistant sublines of murine L1210 leukaemia and B16-BL6 mouse melanoma have been previously described (Ganapathi et al., 1987, 1988; Ganapathi & Grabowski, 1988). Briefly, these sublines were developed by adapting cells to grow continuously in the presence of $0.05\,\mu g\,ml^{-1}$ and $0.25\,\mu g\,ml^{-1}$ DOX and are identified as L1210/DOX0.05, L1210/DOX0.25, B16-BL6/DOX0.05 and B16-BL6/DOX0.25. Based on *in vitro* cytotoxicity studies using a soft agar colony formation assay, the sublines selected in $0.05\,\mu g\,ml^{-1}$ DOX and $0.25\,\mu g\,ml^{-1}$ DOX were 10-fold and 40-fold DOX resistant, respectively, compared to the corresponding parent sensitive cells (L1210/S or B16-BL6/S).

Parent sensitive (L1210/S) and DOX-resistant sublines (L1210/DOX0.05 and L1210/DOX0.25) of L1210 leukaemia were maintained *in vitro* as suspension cultures using RPMI 1640 medium, supplemented with 25 mM HEPES buffer, 2 mM L-glutamine, 10% fetal bovine serum (FBS), and 10 $\mu$M 2-mercaptoethanol. The doubling time *in vitro* of the L1210 sensitive and DOX-resistant sublines was $10.7 \pm 0.1$ h (Ganapathi & Grabowski, 1988). The parent-sensitive (B16-BL6/S) and DOX-resistant sublines (B16-BL6/DOX0.05 and B16-BL6/DOX0.25) of B16-BL6 melanoma were maintained *in vitro* as monolayer cultures using Eagle's minimum essential medium with Hank's salts, supplemented with non-essential amino acids, sodium pyruvate, vitamins, 2 mM L-glutamine and 5% FBS. Cells were subcultured weekly using 0.25% trypsin, 0.02% EDTA. The doubling time *in vitro* of the sensitive and progressively DOX-resistant sublines of B16-BL6 melanoma was approximately 16–18 h (Ganapathi et al., 1987, 1988). All *in vitro* cultures were maintained at 37% in a humidified 5% $CO_2$ plus 95% air atmosphere. Media and supplements were obtained from M.A. Bioproducts, (Walkersville, MD, USA) and FBS was from Hyclone Laboratories (Logan, UT, USA).

### *AD 198 and AD 288* in vitro *cytotoxicity*

Cytotoxicity studies were carried out using a soft agar colony-forming assay (Ganapathi & Grabowski, 1988). Log-phase cultures of parent sensitive (L1210/S), 10-fold DOX-resistant (L1210/DOX0.05) and 40-fold DOX-resistant (L1210/DOX0.25) L1210 mouse leukaemia cells at a density of $1 \times 10^6$ cells $ml^{-1}$ in RPMI 1640, supplemented with 10% FBS, were treated with $0.1-2.5\,\mu g\,ml^{-1}$ AD 198 or AD 288 for 3 h at 37°C in a humidified 5% $CO_2$ plus 95% air atmosphere. Cells were centrifuged (80 *g*) and washed twice in drug-free RPMI 1640 supplemented with 10% FBS. Control and treated cells were plated in triplicate at a density of $5 \times 10^3$ cells per 35 × 10 mm Petri dish in RPMI 1640 supplemented with 20% FBS and 10 $\mu$M 2-mercaptoethanol. Following incubation at 37°C in a humidified 5% $CO_2$ plus 95% air atmosphere for 96 h, colonies (>50 cells) in untreated control and treated plates were counted in an Omnicon Feature Analysis System II (Bausch & Lomb, Rochester, New York). Cytotoxicity in sensitive (B16-BL6/S) and progressively DOX-resistant sublines (B16-BL6/DOX0.05 and B16-BL6/DOX0.25) of B16-BL6 melanoma was determined by treating log-phase monolayer cultures *in vitro* with $0.1-2.5\,\mu g\,ml^{-1}$ AD 198 for 1 h at 37°C in a humidified 5% $CO_2$ plus 95% air atmosphere. Cultures were subsequently washed twice with drug-free medium and incubated for an additional 72 h with fresh medium. Cell counts, following trypsinisation in untreated control and treated cultures, were determined by means of a Coulter counter.

### *Cellular accumulation of AD 198* in vitro

In one set of experiments, log-phase cultures of parent sensitive (L1210/S) and progressively DOX-resistant (L1210/DOX0.05 and L1210/DOX0.25) L1210 mouse leukaemia cells at a density of $1 \times 10^6$ cells $ml^{-1}$ in RPMI 1640 supplemented with 10% FBS were treated with $0.25\,\mu g\,ml^{-1}$, $0.5\,\mu g\,ml^{-1}$ and $1.0\,\mu g\,ml^{-1}$ AD 198 at 37°C in a humidified 5% $CO_2$ plus 95% air atmosphere. Duplicate aliquots ($1 \times 10^6$ cells per sample), removed at the end of 1 h of treatment, were centrifuged (100 *g*) and washed twice with 7 ml of ice-cold 0.85% sodium chloride solution. The cells pellet following the final wash was resuspended in 50% ethanol, 0.3N hydrochloric acid, mixed thoroughly in a vortex mixer and centrifuged (700 *g*), and the total anthracycline content in the supernatant was determined fluorimetrically in an Aminco-Bowman spectrophotofluorometer (American Instruments Co., Silver Springs, MD) at excitation and emission wavelengths of 470 nm and 585 nm, respectively (Ganapathi et al., 1984, 1985). Standard curves of AD 198 prepared in 50% ethanol, 0.3N hydrochloric acid were used for computation of total anthracycline content, which was expressed as nmol AD 198-equivalents per $10^6$ cells.

Cellular anthracycline accumulation was also determined by HPLC/flow fluorescence analysis following 3 h drug exposure as part of a detailed study on drug uptake, retention and biotransformation. As above, L1210/S, L1210/DOX0.05 and L1210/DOX0.25 cells were exposed to AD 198 at their $IC_{50}$ concentrations (0.5, 0.5, $1.0\,\mu g\,ml^{-1}$, respectively). After 3 h incubation, the cells were harvested and washed twice with ice-cold 0.85% sodium chloride solution, and the resulting cell pellets were sonicated in cold 0.85% sodium chloride solution (2 ml). For analysis, samples, in duplicate, were diluted with Tris buffer (0.05 M, pH 8.5, 3 ml) and subjected to organic extraction (ethyl acetate: 1-propanol, 9:1 by volume, 2 × 8 ml). The two organic extracts for each sample were combined and evaporated to dryness (37°C) under a stream of dry nitrogen gas. Dried samples were stored at −70°C pending analysis. The efficiency of extraction was monitored by reference to standard curves derived from the extraction of known quantities of authentic standards added to sonicates of untreated cells.

Samples were reconstituted in methanol (60–100 $\mu$l) before assay. HPLC separation conditions were as follows: reverse-phase 10 $\mu$ phenyl-RADIALPAK radial compression column (Waters Associates, Milford, MA); initial mobile phase conditions, 25% acetonitrile: 75% ammonium formate buffer, 0.05 M, pH 4.0; final conditions, 75% acetonitrile: 25% buffer, linear gradient over 20 min; flow rate 2.5 ml $min^{-1}$. Column eluate was monitored by flow fluorimetry (Model FS 970, Schoeffel Instruments, Ramsay, NJ), 482 nm excitation wavelength, 550 nm emission filter. Fluorescent signals were identified and quantified by reference to standard curves constructed from authentic reference samples. Results of HPLC fluorimetric assays are expressed on the basis of $10^6$ viable cells. The *in vitro* and *in vivo* biotransformation products of AD 198 have been rigidly characterised in previous studies (Israel & Seshadri, 1986; Israel *et al.*, 1987*a*, *b*, 1989).

*Cellular retention and biotransformation of AD 198* in vitro

Parental sensitive (L1210/S) and DOX-resistant cells (L1210/DOX0.05 and L1210/DOX0.25) in RPMI 1640 supplemented with 10% FBS were pretreated with the $IC_{50}$ of AD 198 ($0.5 \mu g ml^{-1}$ for L1210/S and L1210/DOX0.05, and $1.0 \mu g ml^{-1}$ for L1210/DOX0.25) for 3 h at 37°C in a humidified 5% $CO_2$ plus 95% air atmosphere. Cells were then centrifuged, resuspended in drug-free medium (RPMI 1640 supplemented with 10% FBS) and incubated at 37°C. Aliquots of cells ($5 \times 10^6$ cells per sample) from replicate experiments retrieved at the end of the 3 h accumulation phase, and subsequently at 1, 2, 4, 8 and 24 h during the retention phase, were centrifuged (100 *g*) and washed three times in ice-cold 0.85% sodium chloride. Cell pellets were sonicated and extracted, and the levels of AD 198 and metabolites were quantified by HPLC/flow fluorescence detection. Total cellular anthracycline content at each time point was computed by summing the amounts of the individual anthracycline species present; negligible amounts of drug were lost in the wash.

*Antitumour activity of AD 198* in vivo

Female DBA/2n or $CD2F_1$ mice for L1210 mouse leukaemia and male C57Bl/6NCr mice for B16-BL6 mouse melanoma were used for *in vivo* studies. Mice were obtained from the Animal Genetics and Production Branch, Frederick Cancer Research Facility, National Cancer Institute, Fort Detrick, Maryland. Tumour inoculum for L1210 mouse leukaemia and B16-BL6 mouse melanoma was $10^5$ cells and $10^6$ cells, respectively. Cells from log-phase cultures of parental sensitive and DOX-resistant sublines as a single cell suspension (viability >95% based on trypan blue dye exclusion) in sterile 0.9% sodium chloride solution were injected i.p. into mice in groups of six to eight matched for age, weight and sex (day 0). Drugs were formulated for assay in 20% Diluent 12, 80% saline. Groups of mice were subsequently treated i.p. once daily on days 1–4 with the following: (a) vehicle alone (controls); (b) $3 mg kg^{-1}$ DOX; or (c) $10 mg kg^{-1}$ or $12.5 mg kg^{-1}$ of AD 198. The doses of DOX and AD198 used in these studies were based on the optimal doses for these compounds determined in earlier studies (Israel *et al.*, 1975, 1985, 1986, 1987*a*). Each experiment was replicated at least twice for the determination of mean survival time and median per cent increase in lifespan (% ILS) of treated animals relative to untreated controls.

### Results

Cytotoxic effects *in vitro* of AD 198 following drug treatment for 3 h in sensitive and progressively DOX-resistant sublines of L1210 mouse leukaemia are outlined in Figure 1. The data demonstrate that >90% cell kill can be achieved over the range of concentrations evaluated. Further, based on regression analysis of the dose-response curves, the $IC_{50}$ values for AD 198 in the L1210/S and L1210/DOX0.05 cells were not significantly different (approximately $0.35 \mu g ml^{-1}$). However, the $IC_{50}$ of AD 198 for the L1210/DOX0.25 subline ($1.0 \mu g ml^{-1}$) was significantly different ($P < 0.05$). The cytotoxic effects of AD 198 in sensitive and progressively DOX resistant B16-BL6 cells (Figure 2) followed a similar trend, and the $IC_{50}$ was found to be $0.3 \mu g ml^{-1}$ AD 198 for the B16-BL6/S and B16-BL6/DOX0.05 cells, and $1.0 \mu g ml^{-1}$ AD 198 for the B16-BL6/DOX0.25 cells.

Since it is widely accepted that reduced cellular drug uptake and/or retention of anthracyclines is a major mechanism involved in the expression of the MDR phenotype (Inaba *et al.*, 1979; Gerlach *et al.*, 1986*b*), we evaluated both uptake and retention of AD 198 in sensitive and progressively DOX-resistant L1210 mouse leukaemia cells. In a preliminary study, total anthracycline cellular accumulation was determined spectrophotometrically after 1 and 3 h of continuous exposure to AD 198 at three different concentrations (Table I). From results outlined in Table I, it is evident that drug accumulation is dependent on extracellular concentration but independent of time of exposure (1 h *vs* 3 h). Further, unlike results with DOX where differences were seen (Ganapathi & Grabowski, 1988), accumulation of AD 198 was comparable in the sensitive and resistant sublines. In another study, HPLC analytical data were used to determine intracellular anthracycline content after 3 h exposure to the $IC_{50}$ concentrations of AD 198. Data provided in Table II additionally document the extent of drug uptake by cells exposed to AD 198. In this, as in related studies (Israel *et al.*, 1987*b*, 1989), AD 198 was found to accumulate rapidly and extensively in cells, achieving a cell:medium partition ratio of 7:3.

The putative role of P-glycoprotein, which is overexpressed

**Figure 1** Cytotoxicity of *N*-benzyladriamycin-14-valerate (AD 198) in sensitive (–, L1210/S) and progressively doxorubicin-resistant (---, L1210/DOX 0.05; ...., L1210/DOX 0.25) L1210 mouse leukaemia cells. Bars are s.e.

**Figure 2** Cytotoxicity of *N*-benzyladriamycin-14-valerate (AD 198) in sensitive (–, B16-BL6/S) and progressively doxorubicin-resistant (---, B16-BL6/DOX 0.05; ...., B16-BL6/DOX 0.25) B16-BL6 mouse melanoma cells. Bars are s.e.

**Table I** Cellular anthracycline accumulation in sensitive and progressively doxorubicin-resistant L1210 mouse leukaemia cells following continous exposure to AD 198

| *Applied concentration AD 198*[a] | *Time* | *L1210/S* | *L1210/DOX0.05* | *L1210/DOX0.25* |
|---|---|---|---|---|
| 0.25 μg ml$^{-1}$ | 1 h | 0.171[b] | 0.166[b] | 0.163[b] |
| | 3 h | 0.183 | 0.184 | 0.182 |
| 0.5 μg ml$^{-1}$ | 1 h | 0.371 | 0.362 | 0.359 |
| | 3 h | 0.411 | 0.406 | 0.415 |
| 1.0 μg ml$^{-1}$ | 1 h | 0.737 | 0.744 | 0.709 |
| | 3 h | 0.794 | 0.801 | 0.775 |

[a]L1210/S, L1210/DOX0.05 and L1210/DOX0.25 cells were treated with various concentrations of AD 198 and drug content determined fluorimetrically as outlined in **Materials and methods.** Drug concentrations indicated are equivalent to 0.348, 0.697 and 1.393 nmol AD 198 per ml, respectively, [b]Total cellular anthracycline content expressed as nmol AD 198-equivalent per $10^6$ cells. Data are mean values from at least duplicate experiments and the coefficient of variation was <10%.

**Table II** Drug accumulation, retention, and biotransformation in parent sensitive and progressively doxorubicin-resistant L1210 mouse leukaemia cells exposed to AD 198[a]

| *Time* | *L1210/S* | | | *L1210/DOX0.05* | | | *L1210/DOX0.25* | | |
|---|---|---|---|---|---|---|---|---|---|
| | *AD 198* | *AD 288* | *Total anthracycline content*[c] | *AD 198* | *AD 288* | *Total anthracycline content*[c] | *AD 198* | *AD 288* | *Total anthracycline content*[c] |
| *Accumulation*[b] | | | | | | | | | |
| 3 h | 0.384 | 0.018 | 0.492 (71%)[d] | 0.357 | 0.114 | 0.471 (68%)[d] | 0.807 | 0.240 | 1.047 (75%)[d] |
| *Retention*[e] | | | | | | | | | |
| 1 h | 0.212 | 0.088 | 0.300 (61%)[f] | 0.171 | 0.082 | 0.253 (54%)[f] | 0.448 | 0.211 | 0.660 (63%)[f] |
| 2 h | 0.224 | 0.117 | 0.343 (70%) | 0.181 | 0.104 | 0.288 (61%) | 0.448 | 0.228 | 0.684 (65%) |
| 4 h | 0.199 | 0.125 | 0.324 (66%) | 0.154 | 0.120 | 0.286 (61%) | 0.386 | 0.258 | 0.644 (61%) |
| 8 h | 0.171 | 0.150 | 0.327 (66%) | 0.131 | 0.141 | 0.276 (58%) | 0.337 | 0.313 | 0.666 (64%) |
| 24 h | 0.067 | 0.205 | 0.272 (55%) | 0.042 | 0.137 | 0.181 (38%) | 0.159 | 0.303 | 0.479 (46%) |

[a]Cellular AD 198, AD 288, and AD 298 content, as determined by HPLC/flow fluorescence assay, expressed as nmol per $10^6$ cells. Data are mean values from at least duplicate experiments, with a coefficient of variation of <10%. [b]L1210/S and L1210/DOX0.05 cells were treated with 0.5 μg ml$^{-1}$ AD 198 (equivalent to 0.697 nmol per $10^6$ cells per ml) and L1210/DOX0.25 cells were treated with 1.0 μg ml$^{-1}$ AD 198 (equivalent to 1.393 nmol per $10^6$ cells per ml) for 3 h at 37°C. [c]Parent drug plus biotransformation products AD 288 and AD 298; no other anthracycline materials detected. [d]Numbers in parentheses, for the accumulation phase, represent the per cent of the applied drug concentration partitioned into the cells at the end of 3 h of continuous drug exposure. [e]Time following the placement of 3-h pretreated cells in drug-free media. [f]Numbers in parenthesis, for the retention studies, represent the per cent of total anthracycline retained in the cells at the indicated times relative to the amount present after the 3-h accumulation phase.

in the DOX-resistant L1210 cells (Ganapathi *et al.*, 1989), is suggested to involve active drug efflux (Gerlach *et al.*, 1986*b*). Accordingly, in order to correlate cytotoxic effects with drug retention, cells were pretreated with the experimentally determined $IC_{50}$ concentrations of AD198 and retention was determined over 24 h. Retention data for AD 198, provided in Table II, indicate that approximately 40% of presumably loosely bound drug is lost within the first hour when pretreated cells are placed in drug-free medium. Some re-equilibration of drug occurred for a period of time up to 2 h, and thereafter, for at least 6 h, intracellular drug levels remained essentially unchanged. Significant levels of drug were still present within the cells at 24 h. It is of interest to note that at the $IC_{50}$ of AD198 for L1210/DOX0.25 cells, as expected from the applied concentration, both initial uptake and subsequent retention of cellular AD 198 levels were approximately 2-fold greater than for the L1210/S and L1210/DOX0.05 cells. Retention of AD198, expressed as a percentage of the initial drug uptake, was essentially similar in the sensitive and 40-fold resistant sublines, whereas a somewhat lower level of drug was achieved in the L1210/DOX0.05 subline.

Results from studies on AD 198 biotransformation in L1210/S, L1210/DOX0.05, and L1210/DOX0.25 cells during uptake and retention are included in Table II. The data indicate that during the accumulation phase AD 198 is the predominant species, with AD 288 accounting for approximately 25% of the total drug in each cell line. Although AD 198 remains the predominant species during the early phase of the retention (up to 4 h), levels of AD 288 continue to increase and by 24 h exceed those of parent drug. While intracellular levels of AD 198 were similar in the sensitive and L1210/DOX0.05 resistant subline in the L1210/DOX0.25 cells treated with a 2-fold higher drug concentration, cellular levels of AD 198 and AD 288 were, as expected, correspondingly greater. Levels of AD 298, occasionally detectable, represented at most only 1–3% of the total anthracycline content.

Cytotoxic effects *in vitro* of AD 288 following treatment for 3 h in sensitive and progressively DOX-resistant L1210 mouse leukaemia cells are outlined in Figure 3. In contrast to AD 198, cross-resistance to AD 288 was observed ($IC_{50}$ of AD 288 in L1210/S, L1210/DOX0.05 and L1210/DOX0.25 cells, 0.25, 0.5, and 1.0 μg ml$^{-1}$, respectively).

To determine if *in vitro* results have validity in an *in vivo* setting, the antitumour efficacy of AD 198 compared to DOX was evaluated in mice implanted with the sensitive and progressively DOX-resistant tumours. Results of studies with sensitive and progressively DOX-resistant L1210 mouse leukaemia are outlined in Figure 4. In the L1210/S line, DOX was effective, and survival was more than doubled (104% ILS) compared to the untreated control; approximately 10% long-term survivors (>60 days) were observed in replicate experiments. As previously noted with this tumour (Israel & Seshadri, 1985), AD 198 was more effective than DOX; in these studies a % ILS of 189 was obtained,

**Figure 3** Cytotoxicity of *N*-benzyladriamycin (AD 288) in sensitive (–, L1210/S) and progressively doxorubicin-resistant (---, L1210/DOX 0.05; ....., L1210/DOX 0.25) L1210 mouse leukemia cells. Each point is the mean value of replicate determinations from at least duplicate experiments, with a coefficient of variation of <10%.

and in replicate experiments there were 40% long-term survivors (>60 days). In contrast to the results with L1210/S, DOX was ineffective in the L1210/DOX0.05 and L1210/DOX0.25 cells (% ILS only 22–24). Despite the superior therapeutic efficacy of AD 198 *vs* DOX in the L1210/S system, AD 198 proved ineffective in prolonging survival in both the L1210/DOX0.05 and L1210/DOX0.25 (mean survival time similar to the untreated control).

Similar studies comparing the therapeutic efficacy of DOX and AD 198 in sensitive and DOX-resistant B16-BL6 cells are shown in Figure 5. Therapeutic efficacy of DOX in the B16-BL6/S cells was apparent (55% ILS). However, in contrast to the results with L1210/S (Figure 4), AD 198 was found to be only equiactive with DOX in the B16-BL6/S model (61% ILS and no long-term survivors). Similar to results with the DOX-resistant L1210 sublines, DOX was ineffective against the B16-BL6/DOX0.05 and B16-BL6/DOX0.25 tumour systems *in vivo*. In contrast to the results with the DOX-resistant-L1210 cells, AD 198 retained effectiveness (76% ILS) in the B16-BL6/DOX0.05 model at a level essentially equivalent to that in the parent sensitive B16-BL6/S system. However, with the B16/BL6/DOX0.25 cells, no therapeutic efficacy of AD 198 was present.

### Discussion

Tumour cell resistance and, in particular, the multidrug resistant phenotype have received considerable attention at the experimental and clinical level (Gerlach *et al.*, 1986*b*). Since the identification of P-glycoprotein in MDR cells, and its homology to bacterial active transport proteins, resistance to drugs with diverse mechanisms of action has been attributed to an active drug efflux process (Gerlach *et al.*, 1986*a*). Although this mechanism has gained considerable popularity, at least with anthracyclines, there is actually limited correlation between alteration in drug uptake/efflux and expression of resistance (Ganapathi *et al.*, 1986; Louie *et al.*, 1986; Ganapathi & Grabowski, 1988). The present investigation was undertaken to explore the relationship between cytotoxicity and drug accumulation/retention with a novel lipophilic DOX analogue, which in other *in vitro* studies (Maniar *et al.*, 1988; Sweatman *et al.*, 1988) has demonstrated an ability to circumvent resistance in MDR cells.

Using two sets of parent sensitive and progressively DOX-resistant murine cell lines, the present study shows that, *in vitro*, parental L1210/S and B16-BL6/S cells and their 10-fold DOX-resistant sublines are equally sensitive to AD 198, while their counterpart 40-fold DOX-resistant lines are only 2-fold resistant to the analogue (Figure 1 and Figure 2). Following exposure to AD 198, cells rapidly and extensively take up

**Figure 4** Effect of DOX or AD 198 on survival of mice implanted with L1210/S (sensitive), L1210/DOX0.05 (10-fold DOX-resistant) and L1210/DOX0.25 (40-fold DOX-resistant) L1210 mouse leukaemia cells. Mice were implanted i.p. with $10^5$ tumour cells and treated i.p. with vehicle alone (control), DOX or AD 198. Data presented are the composite from two separate experiments with at least six mice per group.

drug, achieving a 7:3 partitioning ratio between cells and medium at equilibrium (Tables I and II). This is in marked contrast to DOX which, in other studies, accumulates gradually and, at equilibrium, achieves a cell:media partitioning ratio of only 1:11 following cellular drug exposure

**Figure 5** Effect of DOX or AD 198 on survival of mice implanted with B16-BL6/S (sensitive) B16-BL6/DOX0.05 (10-fold DOX-resistant) and B16-BL6/DOX0.25 (40-fold DOX-resistant) B16-BL6 mouse melanoma cells. Mice were implanted i.p. with $10^6$ tumour cells and treated i.p. with vehicle alone (control), DOX or AD 198. Data presented are the composite from two separate experiments with at least six mice per group.

(Traganos *et al.*, 1985; Israel *et al.*, 1987*b*, 1989). Furthermore, with AD 198, no significant difference is seen in drug accumulation between parent sensitive and 10-fold DOX-resistant L1210 cells exposed to their $IC_{50}$ concentrations; 40-fold DOX-resistant L1210 cells, with an $IC_{50}$ twice that of the sensitive and 10-fold DOX-resistant cells, correspondingly accumulate 2-fold higher drug levels under similar conditions.

Intracellular drug persists at high levels in cells which are pretreated with AD 198 at the $IC_{50}$ concentration and then transferred to drug-free medium. While drug efflux was perhaps somewhat greater in the 10-fold DOX-resistant L1210 cells, no major differences were apparent with respect to the extent of drug retention in L1210/S compared to the progressively DOX-resistant sublines with all three cell lines retaining for over 8 h about 60% of the 3 h accumulated drug levels (Table II). Also, no significant differences in cellular drug biotransformation were seen in L1210/S *vs* progressively DOX-resistant cells (Table II).

Like many other MDR model systems (Gerlach *et al.*, 1986*b*), the DOX-resistant sublines of L1210 leukaemia (Ganapathi *et al.*, 1989) and B16-BL6 melanoma (Ganapathi *et al.*, 1987) overexpress P-glycoprotein. However, overall, the results presented here indicate that reduced drug uptake and/or retention *in vitro* is not the determinant of AD 198 cytotoxicity in the resistant cell lines studied. A better correlation exists with cellular drug accumulation required to achieve equivalent cytotoxicity. In this same vein, results from earlier studies with DOX in the progressively DOX-resistant L1210 and B16-BL6 cells also indicated that, for equivalent cytotoxicity, the resistant cells accumulated and retained significantly higher drug levels (Ganapathi & Grabowski, 1988; Ganapathi *et al.*, 1987, 1988).

The present results suggest that the sensitivity of the cellular target responsible for anthracycline cytotoxicity is altered with increasing resistance. In these model systems, no alterations in glutathione metabolism have been observed (unpublished observations), suggesting expression of resistance by alternate mechanisms. One putative target for DOX-induced cytotoxicity involves damage to DNA through drug interference of DNA topoisomerase II activity (Tewey *et al.*, 1984; Silber *et al.*, 1987). Comparative studies in L1210 leukaemia indicate that topoismerase II-mediated DNA cleavage by nuclear extracts is significantly attenuated in the DOX-resistant sublines *vs* the parent sensitive cells (Ganapathi *et al.*, 1989). Although AD 198 and its principal biotransformation product AD 288 cause significant levels of protein-associated DNA strand breaks on alkaline elution assay (Potmesil *et al.*, 1986), neither of these agents can be shown to inhibit isolated mammalian DNA topoisomerase II (Bodley *et al.*, 1989). Thus, AD 198 and intracellularly derived AD 288 may interact with DNA topoisomerase II in a manner quite different from that of DOX, and this difference could be a basis of enhanced sensitivity to AD 198 *in vitro* in DOX-resistant cells.

Based on studies suggesting the role of reduced drug uptake and/or retention as a mechanism of resistance, the calcium modifiers (i.e. calcium blockers and calmodulin inhibitors) are suggested to enhance cytotoxicity in resistant cells by increasing drug retention (Tsuruo *et al.*, 1982; Ganapathi & Grabowski, 1983). However, in studies with the calmodulin inhibitor trifluoperazine (TFP), this agent was previously shown to be ineffective in modulating cellular drug levels and/or cytotoxicity of lipophilic anthracyclines (Ganapathi *et al.*, 1984, 1985). Similarly, in the present study, neither the accumulation nor the cytotoxicity of AD 198 were altered by TFP in the sensitive or DOX-resistant sublines. These results with TFP are distinct from those observed with DOX (Ganapathi & Grabowski, 1988), and suggest that the role of TFP in modulating cytotoxicity of DOX *vs* AD 198 may possibly relate to differences in mechanism of action between these agents. Overall, the present results on cellular drug levels and cytotoxicity of AD 198, and the effects of TFP in progressively DOX-resistant cells (10–40-fold) are in agreement with previous studies on lipophilic anthracyclines in >100 fold DOX-resistant tumour cells (Ganapathi *et al.*, 1984, 1985).

Consistent with earlier *in vivo* observations (Israel *et al.*, 1987*a*; Israel & Seshadri, 1986), AD 198 was again found to be therapeutically superior to DOX in the L1210/S system (Figure 4). However, despite the near or total absence of cross-resistance to DOX seen *in vitro*, no therapeutic benefit was afforded with AD 198 against L1210/DOX0.05 and L1210/DOX0.25 *in vivo* on the daily × 4 treatment schedule. Drug metabolism might be responsible for the treatment failure seen with AD 198 against these DOX-resistant L1210 sublines. Cytotoxicity with AD 198 is considered to be due to the effects of parent drug and its mechanistically different biotransformation product AD 288, which, together, persist at high levels within the cells. The conversion of AD 198 into its principal metabolite AD 288 occurs more rapidly *in vivo* than *in vitro*. Although AD 288 is more cytotoxic than AD 198 *vs* cultured L1210/S cells ($IC_{50}$ 0.25 $\mu g\ ml^{-1}$; Figure 3 *vs* Figure 1), when administered to tumour-bearing animals this agent is effective at a level only equivalent to that of DOX (Israel *et al.*, 1987*a*). Biochemically, AD 288 behaves more like DOX than like AD 198 in its interactions with DNA and it fails to show the membrane-active properites seen with AD 198 (Israel *et al.*, 1987*a*). In the present study, against L1210/DOX0.05 and L1210/DOX0.25 cells, the $IC_{50}$ *in vitro* of AD 288 was 2- and 4-fold greater, respectively, than against L1210/S cells (Figure 3), and this may explain the absence of antitumour activity *in vivo* against the DOX-resistant L1210 sublines.

Studies with the highly disseminated L1210/S and progressively DOX-resistant L1210 sublines point to the need to evaluate solid tumour models *in vivo* to demonstrate potential circumvention of resistance by drugs, and in this regard, antitumour activity *in vivo* of AD 198 with the B16-BL6/S melanoma and its progressively DOX-resistant sublines becomes more informative. AD 198 and DOX are equally effective against B16-BL6/S *in vivo* (Figure 5); the lesser response of these drugs against this tumour compared to L1210/S suggests possible differences in the intrinsic sensitivity of the tumour cells to these agents. However, with the B16-BL6 model system, the activity of AD 198 is retained against the 10-fold DOX-resistant tumour, while DOX is ineffective. Thus, the sensitivity of AD 198 *vs* B16-BL6/DOX0.05 seen *in vitro* is correspondingly achieved *in vivo*. In the 40-fold DOX-resistant subline *in vivo* with AD 198 activity is abolished. Since only a 2-fold level of resistance is seen *in vitro* with AD 198 in the B16-BL6/DOX0.25 (40-fold DOX-resistant) compared to parent B16-BL6/S cells, loss of *in vivo* activity suggests, at least for this system, that a 2-fold difference of $IC_{50}$ *in vitro* may be capable of negating the therapeutic activity of an otherwise superior DOX analogue.

In summary, results from this study demonstratre that in progressively DOX-resistant (<40-fold) model systems, little (<2-fold) or no cross-resistance *in vitro* to AD 198 is observed. Although, unlike DOX (Ganapathi & Grabowski, 1988), the cellular uptake and/or retention of AD 198 is not dependent on the level of resistance, for equivalent cytotoxicity the 40-fold resistant tumours tend to accumulate and retain significantly higher levels of drug. Finally, the limited therapeutic efficacy of AD 198 against DOX-resistant tumours suggests that *in vitro* cellular pharmacokinetics and cytotoxicity studies of novel anthracyclines by themselves may be of limited value in characterising therapeutic efficacy of these agents for the treatment of DOX-resistant tumours *in vivo*. However, the results presented provide an excellent model for further studies to identify the factors in MDR cells which result in resistance to chemotherapy *in vitro* and *in vivo*.

This work was supported in part by research grants CA35531, CA37082, and CA37209 from the National Cancer Institute, US Public Health Service. The authors gratefully acknowledge Dr George W. Williams, Michele Melia and Sharon VanderBrug Medendorp, MPH, Cleveland Clinic Foundation, for statistical analysis of the data; and Sabrina Rashed, University of Tennessee, for skilful preparation of the manuscript.

### References

BLUM, R.H. & CARTER, S.K. (1974). Adriamycin: a new anticancer drug with significant clinical activity. *Ann. Intern. Med.*, **80**, 249.

BODLEY, A., LIU, L.F., ISRAEL, M. & 5 others (1989). *N*-Alkyl analogs of doxorubicin do not inhibit DNA topoisomerase II. *Proc. Am. Assoc. Cancer Res.*, **30**, 621.

CHANDRASEKARAN, B., DIMLING, J. & CAPIZZI, R.L. (1987). Cross-resistance of Menogaril and mitoxantrone in a subline of P388 leukemia resistant to doxorubicin. *Cancer Treat. Rep.*, **71**, 195.

GANAPATHI, R. & GRABOWSKI, D. (1983). Enhancement of sensitivity to adriamycin in resistant P388 leukemia by the calmodulin inhibitor trifluoperazine. *Cancer Res.*, **43**, 3696.

GANAPATHI, R. & GRABOWSKI, D. (1988). Differential effect of the calmodulin inhibitor trifluoperazine in modulating cellular accumulation, retention and cytotoxicity of doxorubicin in progressively doxorubicin-resistant L1210 mouse leukemia cells. Lack of correlation between cellular doxorubicin levels and expression of resistance. *Biochem. Pharmacol.*, **37**, 185.

GANAPATHI, R., GRABOWSKI, D., FORD, J., HEISS, C., KERRIGAN, D. & POMMIER, Y. (1989). Progressive resistance to doxorubicin in mouse leukemia L1210 cells wtih multidrug-resistant phenotype correlates with reductions in drug-induced topoisomerase II-mediated DNA cleavage. *Proc. Am. Assoc. Cancer Res.*, **30**, 529.

GANAPATHI, R., GRABOWSKI, D., ROUSE, W. & RIEGLER, F. (1984). Differential effect of the calmodulin inhibitor trifluoperazine on cellular accumulation, retention and cytotoxicity of anthracyclines in doxorubicin (adriamycin)-resistant P388 mouse leukemia cells. *Cancer Res.*, **44**, 5056.

GANAPATHI, R., GRABOWSKI, D., SCHMIDT, H., SESHADRI, R. & ISRAEL, M. (1985). Calmodulin inhibitor trifluoperazine selectively enhances cytotoxic effects of strong vs weak DNA binding antitumor drugs in doxorubicin-resistant P388 mouse leukemia cells. *Biochem. Biophys. Res. Commun.*, **131**, 912.

GANAPATHI, R., GRABOWSKI, D., SCHMIDT, H., BELL, D. & MELIA, M. (1987). Characterization *in vitro* and *in vivo* of progressively adriamycin-resistant B16-BL6 mouse melanoma cells. *Cancer Res.*, **47**, 3464.

GANAPATHI, R., SCHMIDT, H., GRABOWSKI, D., MELIA, M. & RATLIFF, N. (1988). Modulation *in vitro* and *in vivo* of cytotoxicity but not cellular levels of doxorubicin by the calmodulin inhibitor trifluoperazine is dependent on the level of resistance. *Br. J. Cancer*, **58**, 340.

GANAPATHI, R., YEN, A., GRABOWSKI, D., SCHMIDT, H., TURINIC, R. & VALENZUELA, R. (1986). Role of the calmodulin inhibitor trifluoperazine on the induction and expression of cell cycle traverse perturbation and cytotoxicity of daunorubicin and doxorubicin (adriamycin) in doxorubicin-resistant P388 mouse leukemia cells. *Br. J. Cancer*, **53**, 561.

GERLACH, J.H., ENDICOTT, J.A., JURANKA, P.F. & 4 others (1986*a*) Homology between P-glycoprotein and a bacterial haemolysin protein suggests a model for multidrug resistance. *Nature*, **324**, 485.

GERLACH, J.H., KARTNER, N., BELL, D.R. & LING, V. (1986*b*). Multidrug resistance. *Cancer Surveys*, **5**, 25.

INABA, M., KOBAYASHI, H., SAKURAI, Y. & JOHNSON, R.K. (1979). Active efflux of daunorubicin and adriamycin in sensitive and resistant sublines of P388 leukemia. *Cancer Res.*, **39**, 2200.

ISRAEL, M., MODEST, E.J. & FREI, E. III (1975). *N*-Trifluoroacetyladriamycin-14-valerate, an analog with greater experimental antitumor activity and less toxicity than adriamycin. *Cancer Res.*, **35**, 1365.

ISRAEL, M. & SESHADRI, R. (1986). *N*-Alkyl and *N*-benzyl adriamycin derivatives. US Patent no. 4,610,977, 9 September 1986.

ISRAEL, M., SESHADRI, R. & IDRISS, J.M. (1985). *N*-Benzyladriamycin-14-valerate (AD 198), a promising new adriamycin (ADR) analog. *Proc. Am. Assoc. Cancer Res.*, **26**, 220.

ISRAEL, M., SESHADRI, R., KOSEKI, Y., SWEATMAN, T.W. & IDRISS, J.M. (1987*a*). Amelioration of adriamycin toxicity through modification of drug-DNA binding properties. *Cancer Treat. Rev.*, **14**, 163.

ISRAEL, M., SWEATMAN, T.W., KOSEKI, Y. & SESHADRI, R. (1987*b*). Comparative uptake and retention of adriamycin (ADR) and *N*-benzyladriamycin-14-valerate (AD 198) in cultured CEM cells using complementary radioisotope and HPLC fluorescence assays. *Proc. Am. Assoc. Cancer Res.*, **28**, 264.

ISRAEL, M., SWEATMAN, T.W., SESHADRI, R. & KOSEKI, Y. (1989). Comparative uptake and retention of adriamycin and *N*-benzyladriamycin-14-valerate in human CEM leukemic lymphocyte cell cultures. *Cancer Chemother. Pharmacol.*, (in the press.)

JOHNSON, R.K., CHITNIS, M.P., EMBREY, W.M. & GREGORY, E.B. (1978). *In vivo* characteristics of resistance and cross-resistance of an adriamycin-resistant subline of P388 leukemia. *Cancer Treat. Rep.*, **62**, 1535.

LOUIE, K.G., HAMILTON, T.C., WINKER, M.A. & 8 others (1986). Adriamycin accumulation and metabolism in adriamycin-sensitive and -resistant human ovarian cancer cell lines. *Biochem. Pharmacol.*, **35**, 467.

MANIAR, N., KRISHAN, A., ISRAEL, M. & SAMY, T.S.A. (1988). Anthracycline-induced DNA breaks and resealing in doxorubicin-resistant murine leukemia P388 cells. *Biochem. Pharmacol*, **37**, 1763.

MUGGIA, F.M., YOUNG, C.W. & CARTER, S.K. (eds) (1982). *Anthracycline Antibiotics in Cancer Therapy*, p.3 Martinus Nijhoff: The Hague.

POTMESIL, M., ISRAEL, M., KIRSCHENBAUM, S., SWEATMAN, T.W. & SILBER, R. (1986). Cellular metabolism and DNA interactions of *N*-acyl and *N*-alkylanthracyclines. *Abstracts, 14th Intl. Cancer Congress, Budapest, Hungary*, **1**, 155.

SILBER, R., LIU, L.F., ISRAEL, M. & 6 others (1987). Metabolic activation of *N*-acylanthracylines precedes their interaction with DNA topoisomerase II. *NCI Monogr.*, **4**, 111.

SKOVSGAARD, T. (1978). Mechanisms of resistance to daunorubicin in Ehrlich ascitis tumor cells. *Cancer Res.*, **38**, 1785.

SWEATMAN, T.W., KOSEKI, Y., SESHADRI, R., ISRAEL, M. & BECK, W.T. (1988). Activity of *N*-benzyladriamycin-14-valerate (AD 198) *in vitro* against mechanistically different multidrug-resistant CEM cell lines. *Proc. Am. Assoc. Cancer Res.*, **29**, 271.

TEWEY, K.M., ROWE, T.C., YANG, L., HALLIGAN, B.D. & LIU, L.F. (1984). Adriamycin-induced DNA damage mediated by mammalian DNA topoisomerase II. *Science*, **226**, 466.

TRAGANOS, F., ISRAEL, M., SESHADRI, R., KIRSCHENBAUM, S. & POTMESIL, M. (1985). Effects of new *N*-alkyl analogues of adriamycin on *in vitro* survival and cell-cycle progression. *Cancer Res.*, **45**, 6272.

TSURUO, T., IIDA, M., TSUKAGOSHI, S. & SAKURAI, Y. (1982). Increased accumulation of vincristine and adriamycin in drug-resistant P388 tumor cells following incubation with calcium antagonists and calmodulin inhibitors. *Cancer Res.*, **42**, 4730.

YOUNG, R.C., OZOLS, R.F., & MYERS, C.E. (1981). The anthracycline antineoplastic drugs. *N. Engl. J. Med.*, **305**, 139.